# Synthetic Progestins in Waste and Surface Waters: Concentrations, Impacts and Ecological Risk

**DOI:** 10.3390/toxics10040163

**Published:** 2022-03-29

**Authors:** Maria João Rocha, Eduardo Rocha

**Affiliations:** 1 Laboratory of Histology and Embryology, Department of Microscopy, Institute of Biomedical Sciences Abel Salazar (ICBAS), University of Porto (U.Porto), 4050-313 Porto, Portugal; erocha@icbas.up.pt; 2 Histomorphology, Physiopathology, and Applied Toxicology Team, Interdisciplinary Centre of Marine and Environmental Research (CIIMAR), University of Porto (U.Porto), 4450-208 Porto, Portugal

**Keywords:** drospirenone, EDCs, estranes, gestagens, gonanes, norpregnanes, pregnanes, risk assessment

## Abstract

Synthetic progestins (PGs) are a large family of hormones used in continuously growing amounts in human and animal contraception and medicinal therapies. Because wastewater treatment plants (WWTPs) are unable to eradicate PGs after excretion, they are discharged into aquatic systems, where they can also be regenerated from conjugated PG metabolites. This review summarises the concentrations of 12 PGs in waters from 2015 to 2021. The selected PGs were considered of particular interest due to their wide use, activity, and hormonal derivation (from testosterone, progesterone, and spirolactone). We concluded that PGs had been analysed in WWTPs influents and effluents and, to a lesser extent, in other matrices, including surface waters, where their concentrations range from ng/L to a few µg/L. Because of their high affinity for cell hormone receptors, PGs are endocrine disruptor compounds that may alter the reproductive fitness and development of biota. This review focused on their biological effects in fish, which are the most used aquatic model organisms to qualify the impacts of PGs, highlighting the risks that environmental concentrations pose to their health, fecundity, and fertility. It is concluded that PGs research should be expanded because of the still limited data on their environmental concentrations and effects.

## 1. Introduction

Due to water’s vital importance for life, its availability, quality, and governance have been the subject of intense concern, conflicting interests, and heated debate involving communities, industries, governments, and the media [1]. Nonetheless, past actions and the uncontrolled spread of human activities continue to impact water quality and, more broadly, the vast global aquatic ecosystems [2]. One contemporaneous problem widely recognised as serious for mankind is water pollution, including the increase of the concentrations of compounds defined as micropollutants [3,4].

Water micropollutants are currently mostly anthropogenic in origin and include natural and synthetic compounds that enter the aquatic compartment at concentrations ranging from ng/L to µg/L [5]. Among these contaminants are endocrine disruptor compounds (EDCs). Many of them are active ingredients in hormonal medicines, such as synthetic progestins (also called gestagens, progestogens, or progestins), being of particular concern because they are massively used and designed to act in extremely low dosages in specific cellular receptors [6,7].

In humans, progestins (PGs) are used instead of progesterone in endocrine therapy due to the rapid metabolisation of the latter hormone [8]. These substances are used not only as contraceptives, as PGs can inhibit ovulation and the proliferation of the endometrium, but also to treat and prevent endometrial hyperplasia and carcinoma [9,10], to control dysfunctional uterine bleeding [11], and even to stimulate the appetite of cancer patients [12]. In veterinary medicine and zootechny, these compounds are also used in therapies of cows and mares (viz. in disorders of the reproductive system) and for estrus synchronisation and preparation of donor and receptor animals in cases of embryo transfer [13].

Presently, estimating global PGs usage is challenging due to a lack of data on the issue as well as differences in the types of active pharmaceutical chemicals commonly used by each nation [6]. However, recent data point to this issue as an emerging problem due to the increasing worldwide use of PGs, with the “Progesterone Market” predicting a 13.1% increase over the next five years (https://www.mordorintelligence.com/industry-reports/progesterone-market, accessed on 27 December 2021).

Thus, the widespread use of PGs around the globe and their potential to disrupt non-target organisms in aquatic environments has been considered a hot topic that deserves investigation and timely synthesis reviews [6,7]. This review intends to (a) summarise the properties and the levels of 12 highly prominent synthetic PGs in aquatic environments, covering from 2015 to 2021—i.e., considering the period after the last elaborate reviews on this subject [6,7]—in Section 2 and Section 3; (b) look over the biologic impacts of PGs on fish in Section 4; (c) consider their ability to promote biologic effects similar to those referred in humans in Section 5; and (d) provide hazard coefficients with the objective of prevising possible risks for the aquatic ecosystems exposed to PGs in Section 6.

## 2. Classification and Properties of the Most Prominent PGs in Aquatic Environments

PGs are typically classified considering their structural derivation and “generation” (Table 1). The latter broadly indicates when PGs were introduced to the market. Thus, to understand the effects of PGs, the most relevant classification system is to group them by structure based on the steroid molecule from which they were created; i.e., testosterone, progesterone, and spironolactone [14].

Most of the older PGs were designed during the 1960–1970s and have antigonadotrophic effects [15]. The testosterone derivatives, the “gonanes and estranes”, also referred to as levonorgestrel (LNG) and norethisterone (NTD) families [16], have variate activities (Table 1). The gonanes, such as gestodene (GES), norgestrel (NET), and more specifically, its active stereoisomer levonorgestrel (LNG), have high androgenic effects [17]. In contrast, etonogestrel (ENG), which is the biologically active metabolite of desogestrel, is an agonist of the progesterone receptor (PR), showing low androgenic activity and simultaneous glucocorticoid effects [18].

The estranes, NTD and norethisterone acetate (NTDA), have medium androgenic activity [17]. Dienogest (DIE), classified as a fourth generation progestin, is highly specific for the PR [19] and has no androgenic activity [20]. DIE is usually known as a hybrid progestin, as it has the chemical structure of 19-nortestosterone derivatives but shows antiandrogenic activity characteristics, which are typical of progesterone derivatives [20].

The progesterone derivatives, such as those closely related to 19-norprogesterone, which includes nomegestrol acetate (NOMAC), are called “pure” progestational molecules as they bind almost exclusively to the PR and do not interfere with another steroid receptor [19].

In contrast, those PGs derived from 17-hydroxyprogesterone exhibit varying activities. Thus, medroxyprogesterone acetate (MPA) and its metabolite medroxyprogesterone (MEP) has slight androgenic action and exerts glucocorticoid activity when given at high doses [21]. Megestrol acetate (MGA) has 50% fewer glucocorticoid effects than MPA [15]. These PGs also act in specific areas of the hypothalamus as antiandrogenic molecules [22]. This action control male sexual behaviour and urine marking—typical of several animals [22]. Moreover, while designed as a PR agonist, MPA has a high binding affinity for glucocorticoid receptors [23,24].

Usually, the most recent PGs derived from progesterone are progestational PGs without androgenic, estrogenic, or glucocorticoid activity. These PGs were conceived to mimic the benefits of progesterone without the undesirable effects of older PGs, such as acne, a decrease in high-density lipoprotein cholesterol (HDL-C), or bloating and water retention [15].

Drospirenone (DSP) is an aldosterone antagonist derived from spironolactone. The primary effect of the latter PG is its anti-mineralocorticoid activity, which causes decreased salt and water retention, leading to lower blood pressure and absence of androgenic effects [25]. Additionally, DSP exhibits partial antiandrogenic activity [26]—a property that may counter the adverse impact of androgens on hair growth, lipid fluctuation patterns, and insulin, and the possible influence of body composition in postmenopausal women [26]. Further details about PGs’ cellular targets and biological activities in humans can be found in the literature [27,28,29,30].

Presently, the newer formulations of PGs usually contain more potent progestins such as DIE, ENG, and DSP due to their specificity for PR and lack of androgenic effects [30]. Table 1 summarises those that are focused upon sin this review.

## 3. Waste and Surface Waters Concentrations of Synthetic Progestins

PGs are considered emerging micropollutants in aquatic ecosystems, where they are usually present in concentrations in the order of ng/L. However, accurately knowing their concentrations in waters is crucial since such tiny amounts are potentially harmful to (at least) fish [6,7]. Likely because analysing PGs requires trace analytical methods for their extraction and quantification, the number of studies concerning the environmental levels of these compounds is still scarce and, in a majority, focused on the concentrations of these hormones in influents and effluents from wastewater treatment plants (WWTPs).

**Figure 1:**
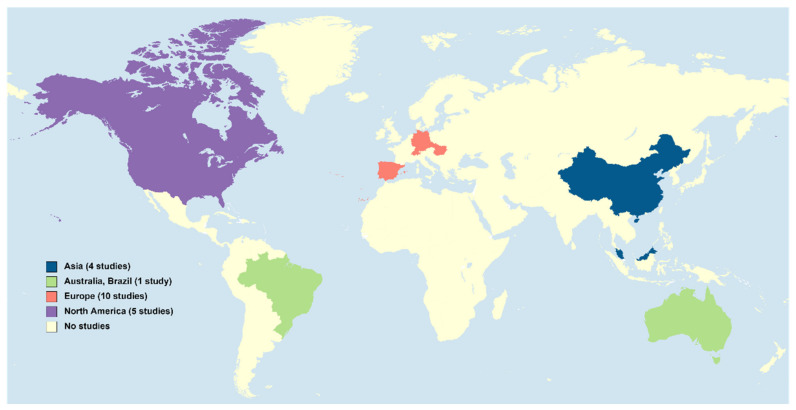
Locations in which studies on the levels of the synthetic PGs considered in this article were conducted in the aquatic environment from 2015 to 2021 (map generated from https://mapchart.net/world.html, accessed on 27 December 2021).

In addition, the surveyed areas are still limited in space (Figure 1). From 2015 to 2021, most publications were performed in Europe (48%) and North America (24%). In Asia (19%), South America (5%), and Australia (5%), there are fewer details about the levels of synthetic PGs, and in Africa, as far as we notice, there are no data on this subject (Figure 1 and Table 2).

Besides, there are also differences concerning the types of PGs analysed. For example, in Europe, the most prevalent PGs in Switzerland [31] were DIE and MPA, whilst in the Czech Republic, it was MGA [32], and in Germany [33], it was DIE. In Asia, a recent study showed LNG, DSP, and dydrogesterone as the most frequently detected PGs in China [34].

**Table 2:** Concentrations of synthetic progestins in waste and surface waters. Average (Av); not detected (ND); not evaluated (n.e.); quantification method (QM); surface waters (S_w_); WWTP influent (WWTP_i_); WWTP effluents (WWTP_e_).

**Testosterone derivatives (Gonanes)**	**PGs**	**QM**	**S_w_** **(ng/L)**	**WWTP_i_ (ng/L)**	**WWTP_e_** **(ng/L)**	**Local (Country)**	**References**
	**GES**	(1)	0.2	3	1	Basel and canton Zürich WWTPs (Switzerland).	[31]
		(2)	<0.05	<0.38–7.7	<0.29–0.71	Blanice River and WWTPs (Czech Republic).	[32]
		(2)	<0.64	<0.41–7.0	<0.19–<3.5	Several WWTPs (Czech and Slovak Republics)	[35]
		(1)	<0.3	n.e.	<1.0	Several WWTPs and rivers (Germany).	[33]
		(3)	<0.2	<3.0	<1.0	Jona River and WWTPs (Switzerland).	[36]
		(4)	<21.5	<21.5	<21.5	Five WWTPs (Portugal).	[37]
	**LNG**	(1)	<2.5–117	493–811	32–39	Langat River Basin (Malaysia).	[38]
		(5)	<2.5	n.e.	<2.5	Southeast Queensland (Australia).	[39]
		(6)	0.85–3.40	n.e.	n.e.	Lake Balaton (Hungry).	[40]
		(7)	<15	n.e.	<15	Two WWTPs in Quebec (Canada).	[41]
		(2)	<0.08	<0.26–<2.1	<0.22–<0.83	Blanice River and WWTPs (Czech Republic).	[32]
		(2)	<0.09	<0.07–<1.2	<0.03–<0.32	Several WWTPs (Czech and Slovak Republics).	[35]
		(1)	<0.05–<0.7	n.e.	<0.3–<1.0	Several WWTPs and rivers (Germany).	[33]
		(1)	ND	ND–38.4	ND–20.1	Several WWTPs, Quebec (Canada).	[42]
		(8)	<2.5	<5–299 ± 17	<3.0	Québec and Ontario (Canada).	[43]
		(4)	n.e.	2.81	1.37	21 WWTPs (China).	[34]
		(4)	n.e.	n.e.	<1.0	Several WWTPs effluents (Germany).	[44]
	**NET**	(4)	n.e.	n.e.	<2.0	Gran Canaria (Spain)	[45]
		(4)	n.e.	11.2	1.92	21 WWTPs (China).	[34]
	**ENG**	(2)	<0.07	<0.28–<1.4	<0.21–<0.89	Blanice River and WWTPs (Czech Republic).	[32]
		(2)	<0.09	<0.25–<1.2	<0.18–<0.94	Several WWTPs (Czech and Slovak Republics).	[35]
		(1)	<0.3	n.e.	<0.5	Several WWTPs and rivers (Germany).	[33]
		(4)	n.e.	n.e.	<1.2	Several WWTPs effluents (Germany).	[44]
**Testosterone derivatives (Estranes)**	**NTD**	(1)	<2.5–230	1048–1137	218–265	Langat River Basin (Malaysia).	[38]
		(4)	n.e.	n.e.	<2.0	Gran Canaria (Spain).	[45]
		(9)	ND–5.20	1.02–94.7 Av. = 25.7	ND–1.68 Av. = 1.25	Four WWTPs, Shanghai (China).	[46]
		(5)	<0.21–3.1	n.e.	n.e.	Freshwater aquaculture (China).	[47]
		(1)	<0.3	<3	<0.6	Basel and canton Zürich WWTPs (Switzerland).	[31]
		(7)	<11	n.e.	<11	Two WWTPs in Quebec (Canada).	[41]
		(2)	<0.04	<0.02–<0.17	<0.03–0.85	Blanice River and WWTPs (Czech Republic).	[32]
		(2)	<0.01	<0.02–<0.91	<0.02–<4.1	Several WWTPs (Czech and Slovak Republics).	[35]
		(1)	n.e.	n.e.	<0.40	Pharmaceutical manufacturing facility discharges (USA).	[48]
		(3)	<0.3	<3	<0.6	Jona River and several WWTPs (Switzerland).	[36]
		(1)	<0.1–<0.3	n.e.	<1.0	Several WWTPs and rivers (Germany).	[33]
		(8)	1.7 ± 0.05–2.7 ± 0.17	<4.8	2 ± 0.2–132 ± 2.2	Québec and Ontario (Canada).	[43]
		(10)	<2.3	<2.3	<2.3	Basque Country (Spain).	[49]
		(1)	ND	ND–78.8	ND–31.8	Several WWTPs, Quebec (Canada).	[42]
		(4)	n.e.	4.02	0.20	21 WWTPs (China).	[34]
		(4)	n.e.	n.e.	<1.0	Several WWTPs effluents (Germany).	[44]
	**NTDA**	(4)	n.e.	10.5	0.24	21 WWTPs (China).	[34]
		(1)	<0.3	n.e.	<0.5	Several WWTPs and rivers (Germany).	[33]
		(4)	n.e.	n.e.	<1.0	Several WWTPs (Germany).	[44]
	**DIE**	(1)	<0.3	<0.8	<0.3	Basel and canton Zürich WWTPs (Switzerland).	[31]
		(3)	<0.3	<0.8	<0.3	Jona River and several WWTPs (Switzerland).	[36]
		(2)	<0.09	1.9–11.0	<0.05–1.0	Blanice River and WWTPs (Czech Republic).	[32]
		(2)	<0.04	1.3–12	<0.04–<4.0	Several WWTPs (Czech and Slovak Republics).	[35]
		(1)	<0.02–2.3	n.e.	1.3–4.4	Several WWTPs and rivers (Germany).	[33]
		(4)	n.e.	n.e.	0.3–3.7	Several WWTPs effluents (Germany).	[44]
**Progesterone derivatives**	**NOMAC**	(2)	<0.07	<0.08–3.6	<0.03–0.26	Blanice River and WWTPs (Czech Republic).	[32]
	**MEP**	(5)	<0.07–1.3	n.e.	n.e.	Freshwater aquaculture (China).	[47]
		(1)	<0.6	<6	<3	Basel and canton Zürich WWTPs (Switzerland).	[31]
		(3)	<0.6	<6	<3	Jona River and several WWTPs (Switzerland).	[36]
		(2)	<0.06	<0.02–<0.13	<0.03–0.23	Blanice River and WWTPs (Czech Republic).	[32]
		(1)	ND	ND–5.7	ND–2.9	Several WWTPs, Quebec (Canada).	[42]
		(2)	<0.04	<0.01–<0.53	<0.01–0.95	Several WWTPs (Czech and Slovak Republics).	[35]
		(1)	<0.05	n.e.	<0.08	Several WWTPs and rivers (Germany).	[33]
	**MPA**	(5)	<0.21–0.31	n.e.	n.e.	Freshwater aquaculture (China).	[47]
		(1)	<0.1	<0.8	<0.2	Basel and canton Zürich WWTPs (Switzerland).	[31]
		(2)	<0.1	<0.15–4.4	<0.09–0.58	Blanice River and WWTPs (Czech Republic).	[32]
		(2)	<0.01	<0.04–8.1	<0.04–0.38	Several WWTPs (Czech and Slovak Republics).	[35]
		(3)	<0.1	<0.8–5.3	<0.2	Jona River and several WWTPs (Switzerland).	[36]
		(1)	<0.05–0.1	n.e.	<0.08–<0.3	Several WWTPs and rivers (Germany).	[33]
		(4)	n.e.	3.09	0.23	21 WWTPs (China).	[34]
		(4)	n.e.	n.e.	<0.6	Several WWTPs effluents (Germany).	[44]
	**MGA**	(4)	n.e.	n.e.	<60	Gran Canaria (Spain).	[45]
		(1)	<0.1	<1	<0.6	Basel and canton Zürich WWTPs (Switzerland).	[31]
		(2)	<0.01	0.52–13.0	0.13–1.0	Several WWTPs (Czech and Slovak Republics).	[35]
		(1)	<0.05–<0.2	n.e.	<0.06–<0.3	Several WWTPs and rivers (Germany).	[33]
		(2)	<0.07	<0.03–<6.3	<0.06–0.4	Blanice River and WWTPs (Czech Republic).	[32]
		(7)	<6–<20	n.e.	n.e.	Water bodies in Santa Maria (Brazil).	[50]
		(4)	n.e.	0.84	0.29	21 WWTPs (China).	[34]
**Spironolactone** **derivative**	**DSP**	(6)	0.26–4.30	n.e.	n.e.	Lake Balaton (Hungry).	[40]
		(1)	<0.3	<4	<1	Basel and canton Zürich WWTPs (Switzerland).	[31]
		(2)	<0.85	0.64–0.77	<0.18–<0.62	Blanice River and WWTPs (Czech Republic).	[32]
		(2)	<0.04	0.34–6.7	<0.07–<0.29	Several WWTPs (Czech and Slovak Republics).	[35]
		(3)	<0.3	<4	<1	Jona River and several WWTPs (Switzerland).	[36]
		(1)	<0.3	n.e.	<0.05	Several WWTPs and rivers (Germany).	[33]
		(4)	n.e.	0.69	0.39	21 WWTPs (China).	[34]
		(4)	n.e.	n.e.	<0.8	Several WWTPs effluents (Germany).	[44]

(1) Liquid chromatography with tandem mass spectrometry detection (LC-MS/MS); (2) liquid chromatography tandem atmospheric pressure chemical ionization/atmospheric pressure photoionization with hybrid quadrupole/orbital trap mass spectrometry operated in high-resolution product scan mode (LC-APCI/APPI-HRPS); (3) high-performance liquid chromatography coupled to a triple quadrupole mass spectrometry (HPLC-MS/MS); (4) ultra-performance liquid chromatography coupled with tandem mass detection (UPLC-MS/MS); (5) gas chromatography with tandem mass spectrometry detection (GC-MS/MS); (6) high-performance liquid chromatography–mass spectrometry (HPLC-MS); (7) liquid chromatography–mass spectrometry (LC-MS); (8) triple quadrupole-linear ion trap mass spectrometer using the sMRM (scheduled multiple reaction monitoring) mode (TripleQuad-LIT-MS); (9) rapid resolution liquid chromatography/tandem mass spectrometry (RRLC-MS/MS); (10) laser diode thermal desorption–tandem mass spectrometry (LDTD–MS/MS).

Here, concerning the 12 PGs in Table 1, the most investigated (%) were NTD (20%) and LNG (14%). There are still less data concerning MPA, DSP (10%), MEP, MGA (9%), GES, DIE (8%), ENG (5%), NTDA (4%), NET (3%), and NOMAC (1%) (Table 2).

Therefore, in an accessible and organised way, this paper compiles the existing data in the bibliography relative to the concentrations of 12 PGs from 2015 to 2021, using the “Web of Science Core Collection” and “PubMed” as primary databases. Thus, Table 2 presents data on the concentrations of these hormones in surface waters and wastewater treatment plants (WWTPs) worldwide, considering their influents and effluents.

Data in Table 2 were gathered from investigations conducted in various geographic locations, with varying PG inputs, and analysed according to well-established analytical techniques, despite the varying detection and quantification levels and accuracies. It is important to stress that some of the surveyed areas in Asia [38,46,47] are densely populated, which may explain the high amounts of PGs measured in surface waters. Therefore, the disparities between studies from distinct regions are not surprising, corresponding to a wide range of concentrations even when including the three compartments of surface waters, WWTP influents, and WWTP effluents.

**Figure 2:**
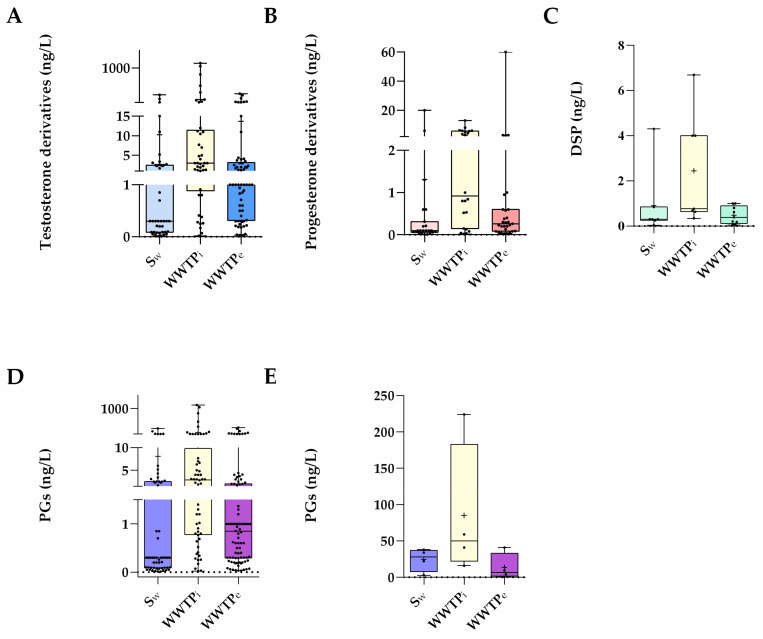
Data are expressed in boxplots with the minimum, median, maximum, average (+), and interquartile range Q1–Q3. Dots represent average individual values measured in surface waters (S_w_), WWTP influent (WWTP_i_) and WWTP effluents (WWTP_e_) around the world concerning PGs derivates from (**A**) Testosterone (n = 42 S_w_, n = 42 WWTP_i_, and n = 62 WWTP_e_), (**B**) Progesterone (n = 23 S_w_, n = 22 WWTP_i_, and n = 29 WWTP_e_), (**C**) Spirolactone (n = 7 S_w_, n = 7 WWTP_i_, and n = 9 WWTP_e_), (**D**) all PGs as a whole (n = 72 S_w_, n = 71 WWTP_i_ and n = 100 WWTP_e_), (**E**) all PGs referred in a previous review (n = 4) [7].

**Figure 3:**
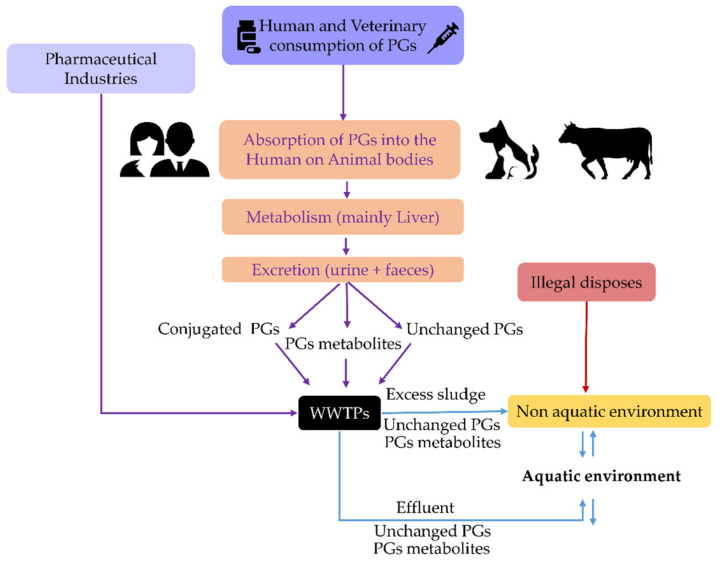
Sources and pathways for the occurrence of progestins in the environment. The distributions of PGs were based on Besse and Garric (2009) [51].

Despite the differences mentioned, Figure 2 shows that synthetic PGs are still present in surface waters in amounts comparable to those observed in WWTP effluents, which is concerning given that dilution is predicted in surface waters. A similar observation was also noticed in previous studies [6,7]. As a result, one can infer that WWTPs do not effectively remove these compounds and/or that some of them can be regenerated in the aquatic environment by deconjugation phenomena (Figure 3).

In particular, Figure 2A shows that PGs derived from testosterone, besides being evaluated in a higher number of studies, were also the hormones with higher concentrations (up to ≅1 µg/L) in the aquatic environments, where their global load reaches ≅97.0% of all PGs considered in Table 2 vs. 2.49% and 0.57% for progesterone and spironolactone derivatives.

Table 2 reveals that in surface waters, the concentrations of PGs derived from testosterone were typically higher for LNG (<0.05–117 ng/L) and NTD (<0.01–230 ng/L) than those for GES (<0.05–21.5 ng/L), DIE (<0.02–2.3 ng/L), and NTDA (<0.3 ng/L) ≅ ENG (<0.07–<0.3 ng/L). Data concerning NET in surface waters were not available.

In WWTP influents, the concentrations of LNG (<0.07–811 ng/L ) and NTD (<0.02–1137 ng/L) were consistently higher than those of GES (<0.38–<21.5 ng/L), DIE (<0.8–12.0 ng/L) ≅ NET (11.2 ng/L) ≅ NTDA (10.5 ng/L), and ENG (<0.28–<1.4 ng/L).

In WWTP effluents, the highest concentrations were measured for LNG (<0.03–39 ng/L), NTD (<0.03–265 ng/L), followed by GES (<0.19–<21.5 ng/L), DIE (<0.04–4.4 ng/L), NET (<2.0–1.92 ng/L), NTDA (0.24–<1.0 ng/L) ≅ ENG (<0.21–<1.2 ng/L).

Progesterone-derived PGs more commonly exist in surface waters in concentrations ca. 17-fold lower (Figure 2B) than those reported above for the testosterone derivatives. Such PGs showed similar concentrations to those of the natural hormone progesterone, which ranged from ND to 13.67 ng/L [38,40] in surface waters and from <0.04 ng/L to 24.8 ng/L [32,42] in WWTP influents. In WWTPs effluents, the levels of those PGs were lower than those of progesterone (ND to 110 ng/L) [32,43]. Despite this, progesterone-derived PGs concentrations in surface waters are comparable to those in WWTP effluents, much as testosterone-derived PGs. Moreover, the two most prevalent progesterone-derived PGs, MGA and MEP, were found in identical amounts in all three aquatic compartments.

Regarding surface waters, the concentrations of MGA (<0.01–<20.0 ng/L) seem higher than those of MEP (<0.04–1.3 ng/L), MPA (<0.01–0.31 ng/L) and NOMAC (<0.07 ng/L).

In WWTP influents, the concentrations of MGA (<0.03–13.0 ng/L) reach higher levels, despite overlapping to some extent with those of MPA (<0.04–8.1 ng/L) and MEP (<0.02–5.7 ng/L), with NOMAC (<0.08–3.6 ng/L) levels being suggestively lower. The occurrence of higher amounts of MEP and MPA, despite being punctual, in surface waters than in WWTP influents is very worrying, stressing the need for more studies concerning this subject.

In WWTP effluents, the highest levels were measured for MGA (<0.06–<60 ng/L), followed by MEP (<0.01–2.9 ng/L), MPA (<0.08–0.58 ng/L), and NOMAC (<0.03–0.26 ng/L).

Finally, considering the spironolactone derivative DSP, it is observed that its concentrations in surface waters (<0.04–4.3 ng/L) were higher than those in WWTP effluents (<0.05–<1.0 ng/L) but lower than those from WWTP influents (0.34–6.7 ng/L) (Figure 2C).

Altogether, this review found (Figure 2D) that the current number of studies on PGs in waters sharply increased when compared with those reported in previous reviews [7] (Figure 2E). In addition, the more recent environmental concentrations of these compounds have risen compared to data published before 2015. Additionally, there are studies on DSP that were not available before [7].

As shown in Table 2, there are parent PGs in surface waters whose origin is unknown. Therefore, it is not established if deconjugation occurs in the aquatic environments and/or if there is a lack of efficient removal of these compounds by WWTPs (Figure 3). As such, we found it helpful to determine PGs removal efficiency in WWTPs. For this purpose, when this information was not available in the bibliography, we used concentrations of PGs in WWTPs influents and effluents reported in Table 2 and inserted them in Equation (1):

(1) $$\mathrm{Removal}\;\mathrm{efficiency}\;\left(\%\right)=\frac{PGinfluent-PGeffluent}{PGinfluent}\times 100$$

Globally, the removal efficiency values of PGs in WWTPs are, on average, 73% (Table 3), which is considered a standard removal percentage for steroids in WWTPs [52,53]. However, at some locations (Table 3, values in bold), the presence of parent compounds was higher in WWTP effluents than in their influents; e.g., GES, LNG, ENG, NTD, DIE and MGA in Czech and Slovak Republics’ WWTPs [32,35]. These negative removal efficiency rates have been explained by the deconjugation of metabolised steroid hormones, including synthetic PGs in WWTPs, which become regenerated free parent steroids (Figure 3) by biodegradation, hydrolysis, and even photolysis [51,52,53,54].

Another important aspect shown in Figure 3 is that, beyond the hypothesised regeneration of parent PGs, active metabolites of these pharmaceuticals also arrive in the aquatic environment. Some of these metabolites are still awaiting their identification and answers about their activity [55]. However, others have already been identified. An example of this is shown by the metabolisation of NTDA, which originates ethinylestradiol (EE_2_), a potent estrogen known to produce endocrine disorders in concentrations as low as a few ng/L [44,56]. Additionally, through side-chain cleavage, the PGs closely related to progesterone by metabolisation can produce potent androgens; e.g., 4-adrostene-3,17-dione, and 5α-dihydrotestosterone [57].

Table 4 shows the most recent advances concerning the human metabolisation of the 12 PGs referred to herein. Thus, further studies involving the parent and the active metabolites of these molecules should be considered in future monitoring programs once it is already established that the latter can also induce health disorders in aquatic organisms [57].

## 4. Biological Effects of PGs in Aquatic Organisms, Particularly Fishes

In invertebrates, progesterone also plays a central role in reproduction [73,74]. An ancient origin of progesterone and its receptor is well shown in a study using the micro invertebrate *Brachionus manjavacas* (Rotifera) [73]. This work undoubtedly exemplifies that progesterone and its receptor exhibit conservation of function over a broad range of animals across phylogenies, presenting further evidence about the ancient origin of hormonal steroid regulation and suggesting that the endocrine regulation of mammalian reproduction may be derived from primitive regulatory pathways [73].

In amphibians, natural PGs are also involved in numerous biological activities, which includes gonadal development/differentiation, germinal vesicle breakdown (GVBD), and adequate homeostasis of the hypothalamic–pituitary–gonadal axis (HPG), among others [75].

Compared with tetrapods, the plasma levels of progesterone in fish are usually low, as the hormone is essentially an intermediate in the steroidogenic pathway of these organisms and other natural PGs predominate, such as 17α-hydroxyprogesterone (17-OHP), 17,20β-dihydroxypregn-4-en-3-one (17,20β-P) and 17,20β,21-trihydroxypregn-4-en-3-one (17,20β-S) [76]. These and other PGs are involved in reproductive functions such as follicular steroidogenesis, spermatogenesis, pheromone synthesis, in the homeostasis of the immune and cardiovascular systems, and even as neuroprotectors [76,77,78,79,80,81].

**Figure 4:**
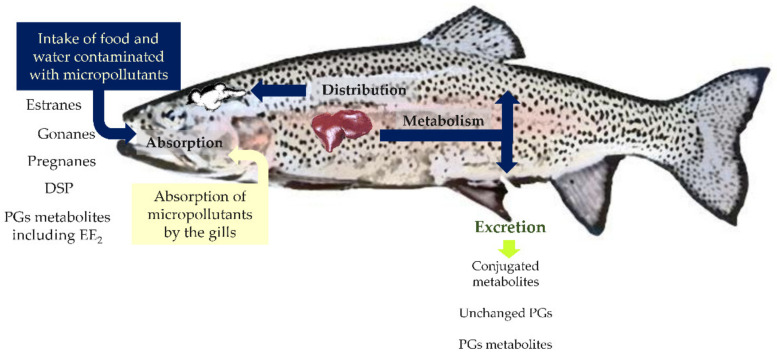
Routes of entry, circulation, major places of action, and the fate of progestins such as pregnanes, estranes, gonanes, spironolactone derivatives, and other EDCs.

These observations demonstrate that PGs are essential steroid hormones of aquatic organisms [76]. Thus, it is not surprising that once in the water, synthetic PGs, when uptaken by animals via food, water ingestion, or direct contact with gills, interact with cell PGs receptors of these organisms, disrupting their normal physiological status (Figure 4).

Therefore, and according to the National Institute of Environmental Health Sciences (NIEHS), PGs are EDCs, as this classification embraces natural or man-made compounds that can mimic or interfere with the function of hormones in an organism, producing a variety of adverse effects on, e.g., reproductive, neurological and immune systems [82].

It was previously stressed that concentrations as low as 0.8–1.0 ng/L of PGs induce endocrine disturbances in fish [6], which falls within the range observed in waters referred to in Table 2. This fact makes PGs one of the most critical pharmaceutical compounds of concern after EE_2_, which can be a metabolisation product of NTDA [33]. The latter estrogen is already included in the EU watchlist of substances with environmental interest [83].

Once the deleterious role of PGs in fish [6], amphibians (Xenopus laevis and X. tropicalenses) [84,85], and even mussels (Dreissena polymorpha) [86] has been recognised, as they interfere with the normal function of vital organs and produce sufficiently important alterations that impact survival, growth, and reproduction [6,75,87,88], more studies involving fish as test organisms have been published. Therefore, we elaborated Table 5 to summarise the advances on the effects of PGs in fish as published from 2015 to 2021.

## 5. Bioconcentration Factors and Predicted Effect Concentrations of PGs in Fish Plasma

Another critical aspect of PGs in fish and other aquatic organisms is that after absorption, the substances can be bioconcentrated, bioaccumulated, or both (Figure 4).

Bioconcentration factors (BCFs) describe the readiness of chemicals to concentrate in organisms when they are present in the environment. These are determined by the ratio between the concentration of a specific chemical inside biological tissues and its levels in the surrounding environment [131]. In vivo experiments led to the obtention of the BCFs for three PGs: the LNG 17–53 [132], NET 2.6–40.8 [133,134], and MPA 4.3–37.8 [135].

Nonetheless, when in vivo assays are not available, it is possible to use “log K_ow_—based models” to assess the BCFs [6]. In fact, BCF values have already been used to predict critical environmental concentrations of 500 pharmaceuticals [6]. Thus, when plasma concentrations of a specific PG and BCFs (either measured or predicted) are known, the environmental concentration of progestins in the surrounding water for fish can be back-calculated.

So, using this method, the BCF_FP_ = bioconcentration factor in fish plasma can be calculated by applying Equation (2) [136,137,138]:

(2) $$\log BCF_{FP}=0.73\times \mathrm{logKow}-0.88$$

In addition, the “Predicted Effect Concentration” (PEC_w_) of a particular chemical can be taken from the bibliography or from in vivo experiments [6,139,140,141]. For instance, when exposing rainbow trout (*Oncorhynchus mykiss*) to 1 ng/mL of LNG, a maximum of 12 ng/mL of this compound existed in plasma, which is a concentration that exceeds five times the human therapeutic dosage of 2.4 ng/mL [136]. In this case, LNG is likely to produce effects in fish in line with those seen in humans. Alternatively, PEC_w_ can be calculated mathematically (3). This approach is based on the concept that when the concentration in the plasma of a fish (C_FP_) reaches the “therapeutic dose” observed in humans, similar effects, at least to some extent, can be expected in fish. This perspective is grounded on the fact that many receptors and enzyme systems are conserved across mammalian and non-mammalian species, making mechanism of action extrapolations possible for a particular compound, considering its environmental concentrations [6,137].

(3) $$PEC_{w}=\frac{C_{FP}}{BCF_{FP}}$$

Here, the values of BCF_FP_ and PEC_w_ for the surveyed PGs were calculated as shown above and used to assess possible deleterious effects of those chemicals in fish (Table 6) beyond their possible bioaccumulation in these organisms [6].

When comparing the BCF_FP_ determined in vivo with those reported in Table 6, it is observed that the current data are consistent with the results obtained for LNG and NET using the channel catfish (*Ictalurus punctatus*), fathead minnow (*P. promelas*) [133,134], and roach (*R. rutilus*) [132]. Specifically, in vivo, the BCFs for the latter two PGs ranged from 2.6 to 40.8, similar to the mathematically estimated BCF_FP_ of 46 (Table 6). However, this mathematical approach is not always comparable with the in vivo assays, as shown by the data obtained for MPA in carp (*C. carpio*) [135]. The previous studies revealed that BCFs for MPA range from 4.3 to 37.8, whereas those determined in Table 6 point to 128.

Thus, regardless of the utility of the theoretical assessments, studies exposing different fish species to the current PGs are needed to avoid inaccurate conclusions that may derive from those kinds of estimates.

Taking this in mind, but still relying on the data in Table 6, it is probable that both LNG and NET exist in surface waters and WWTPs influents in amounts that can induce fish endocrine disruption, as suggested both the theoretically calculated and the in vivo data for these two PGs. However, further studies involving in vivo assays are required to prove indubitably this hypothesis.

Ultimately, when humans eat PGs-contaminated fish, they are unwittingly exposed to these chemicals and thus, at least in theory, human health could be impacted by these contaminants. However, as far as we noticed, there are no published data about this issue.

## 6. Evaluation of Risk Quotients (RQs) for PGs

Because several PGs showed potential to induce adverse effects in fish, it was considered opportune to investigate their impact by examining their risk quotients (RQs) in the aquatic environment. The parameter RQ is known to realistically estimate the potential ecological risk; i.e., the probability of an expected effect or potential danger caused by an environmental concentration of a pollutant.

The calculus of this quotient involves the ratio between the “Measured Environmental Concentrations (MECs)” and the “Predicted No Effect Concentrations (PNECs)” of a certain compound (4) [141,142]:

(4) $$\mathrm{RQ}\;=\frac{MEC}{PNEC}$$

The MECs in this study were the average environmental concentrations provided in Table 2 for surface waters, and the ranking criteria used were RQ > 1.0 for high ecological risk, 0.1 < RQ < 1.0 for medium risk, and RQ < 0.1 for low risk [143]. The use of maximum concentrations and ranking criteria could estimate extreme worst-case scenarios.

The PNECs reflect the relative toxicity of each molecule for fish, and when they are not published, they can be derived by following the standard scientific assessment procedures defined in the EU Guidance for REACH implementation [144]. In those situations, the PNECs can be calculated considering several endpoint values found in the literature or using the “Species Sensitivity Distribution” (SSD) method, divided by their respective “Assessment factors” (AF), as proposed in Table 7 [145].

The preferred endpoints for screening-level risk assessments are values of chronic toxicity tests, represented by the “No Observed Effect Concentration” (NOEC). Whenever NOECs are not accessible, it is also adequate to use the “Lowest Observed Effect Concentration” (LOEC), the median “Effective Concentration” (EC_50_), or the Lethal Concentration (LC_50_) taken from acute toxicity tests.

Although the PNECs of GES, ENG and NTDA were calculated considering acute toxicity tests, those for NOR, NOMAC, and MPA were based on data from chronic toxicity tests in fish. Thus, some of the present conclusions will be valid for chronic while others for acute exposures.

The calculation of the RQs values (Table 8) reveals that the categories of PGs posing a higher ecologic risk for fish are those structurally related to testosterone. All the analysed PGs of this category, except for DIE and NET, show extremely high RQ values. This observation can be related to the extensive usage of these compounds and the higher number of published data considering these compounds vs. the other PGs (i.e., most of the studies involved the measurement of LNG ≅ 40 %, followed by NTD ≅ 18 % and DSP ≅ 16%). It is stressed that both LNG and NET have been referred to in the last section as having the ability to attain or even surpass in fish plasma the therapeutical dosages used in humans (Table 6).

In opposition, almost all PGs structurally related to progesterone and DSP showed RQs < 1, suggesting that these EDCs are less problematic than those referred to above. However, this hypothesis needs further investigation as the number of studies involving these compounds is lower than those for gonanes and estranes.

A final word of caution is due because the estimated risks are for single progestins. To precisely apprehend the global impact of these EDCs on aquatic organisms, research using mixtures and, therefore, better replicating environmental conditions are required. In this regard, there are still very few studies addressing the effects of mixtures of PGs in aquatic animals, covering only a few PGs and a couple of fish species [121,123]. As such, it is premature and would be incorrect to take a mixture toxicology approach.

## 7. Final Remarks

Progestins are confirmed EDCs for aquatic organisms—in particular, for fish living in polluted environments. However, when compared to other pollutants in the same category, such as EE_2_, it can be concluded that PGs are still understudied in terms of their functional effects on aquatic organisms at different trophic levels.

Except for NET, which has not been examined, all other synthetic PGs studied are present in surface waters, and all occur in WWTP influents and effluents. The latter two matrices have been the primary focus of environmental monitoring. As a result, there is a need for other aquatic matrices (e.g., lakes, estuaries, seashores, subterranean waters) to be investigated. In parallel, there is a need to widen the monitoring to more geographic areas, as the majority of studies have been conducted in Europe, Canada, and China.

In our view, the state of art already calls for regulation on the concentration limits for the discharge of PGs in WWTP effluents. Considering the precautionary principle, the pertinence of that possibility should be taken into account in future WFD updates.

Furthermore, because most PG metabolites remain biologically active, their proper identification should be evaluated, and their hazardous impact should be included in future investigations. To fully appreciate the influence of these chemicals in aquatic systems, it is also necessary to examine the biological consequences of complex combinations of parent PGs and their active metabolites.

## Figures and Tables

**Table 1:** Pharmacological groups of the selected progestins referred to in this article considering their structural derivation, generation, and androgenic effects in humans: (+++) highly androgenic; (++) medium androgenic; (+) low androgenic; (-) no androgenic effects.

**Testosterone derivatives**	**Hormone**	**Family**	**Common Name (Acronym) & CAS**	**Structure** **& Molecular Formula**	**Generation** **& Activity**
	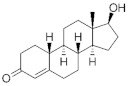 **19-Nortestosterone**	Gonanes (C_17_) 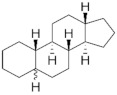 or LNG family	Gestodene (**GES**) 60282-87-4	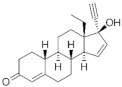 C_21_H_28_O_2_	3rd Generation 1986 (+++)
			Levonorgestrel (**LNG**) 797-63-7	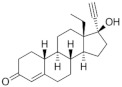 C_21_H_28_O_2_	2nd Generation 1966 (+++)
			Norgestrel (**NET**) 6533-00-2	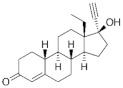 C_21_H_28_O_2_	2nd Generation 1966 (+++)
			Etonogestrel (**ENG**) 54048-10-1	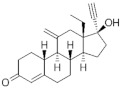 C_22_H_28_O_2_	3rd Generation 1998 (+)
		Estranes (C_18_) 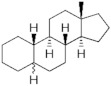 or NTD family	Norethisterone (**NTD**) 68-22-4	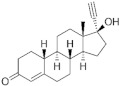 C_20_H_26_O_2_	1st Generation 1951 (++)
			Norethisterone acetate (**NTDA**) 51-98-9	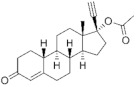 C_22_H_28_O_3_	1st Generation 1951 (++)
			Dienogest (**DIE**) 65928-58-7	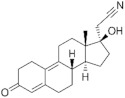 C_22_H_28_O_2_	4th Generation 1978 (-)
**Progesterone derivatives**	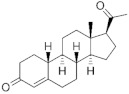 **19-Norprogesterone**	Norpregnanes (C_20_) 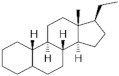	Nomegestrol acetate (**NOMAC**) 58652-20-3	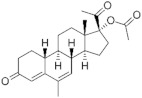 C_23_H_30_O_4_	4th Generation 1986 (-)
	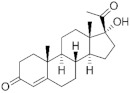 17α-Hydroxyprogesterone	Pregnanes (C_21_) 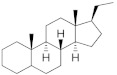	Medroxyprogesterone (**MEP**) 520-85-4	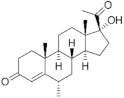 C_22_H_32_O_3_	1st Generation 1957 (+)
			Medroxyprogesterone acetate (**MPA**) 71-58-9	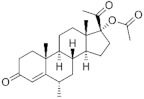 C_24_H_34_O_4_	1st Generation 1957 (+)
			Megestrol acetate (**MGA**) 595-33-5	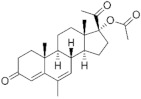 C_22_H_30_O_4_	1st Generation 1963 (-)
**Spironolactone** **derivative**	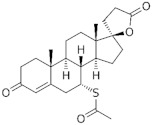 Spironolactone		Drospirenone (**DSP**) 67392-87-4	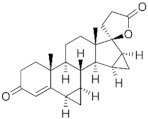 C_24_H_30_O_3_	4th Generation 1976 (-)

**Table 3:** Worldwide WWTPs removal efficiency of PGs. In bold are shown the situations when the treated effluent contains higher amounts of a certain PG than its influent (these values were set apart from the global average percentage of removal, % R_Av._). Quantification method (QM); WWTP influents (WWTP_i_); WWTP effluents (WWTP_e_); % Removal (% R).

**Testosterone derivatives (Gonanes)**	**PGs**	**QM**	**WWTP_i_ (ng/L)**	**WWTP_e_** **(ng/L)**	**% R**	**% R_Av._**	**Local**	**References**
	**GES**	(1)	<3.0	<1.0	66.7	82.9	WWTPs (Switzerland).	[31]
		(2)	6.6	0.5	92.8		WWTPs (Czech Republic).	[32]
		(2)	4.3	0.77	95.5		WWTPs (Czech and Slovak Republics).	[35]
		(3)	3.0	1.0	66.7		WWTPs (Switzerland).	[36]
		(2)	<0.38	<0.49	−28.9	**−186**	WWTPs (Czech Republic).	[32]
		(2)	<0.79	<3.5	−343		WWTPs (Czech and Slovak Republics).	[35]
	**LNG**	(1)	652	35.5	94.4	62.4	WWTPs (Malaysia).	[38]
		(2)	<1.8	0.5	72.4		WWTPs (Czech Republic).	[32]
		(2)	0.43	0.20	54.3		WWTPs (Czech and Slovak Republics).	[35]
		(1)	58.6	26.0	53.7		WWTPs (Canada).	[42]
		(4)	*Data from bibliography*		37.0		WWTPs (China).	[34]
		(2)	<0.26	<0.53	−103.8	**−104**	WWTPs (Czech Republic).	[32]
	**NET**	(4)	*Data from bibliography*		96.0	96.0	WWTPs (China).	[34]
	**ENG**	(2)	1.3	0.6	58.7	42.9	WWTPs (Czech Republic).	[32]
		(2)	0.52	0.38	27.1		WWTPs (Czech and Slovak Republics).	[35]
	**NTD**	(1)	1093	242	78.0	75.7	WWTPs (Malaysia).	[38]
		(6)	*Data from bibliography*		98		WWTPs (China).	[46]
		(1)	<3.0	<0.6	80.0		WWTPs (Switzerland).	[31]
		(2)	0.14	0.08	39		WWTPs (Czech and Slovak Republics).	[35]
		(3)	<3.0	<0.6	80.0		WWTPs (Switzerland).	[36]
		(1)	4.8	2.0	58.3		WWTPs (Canada).	[42]
		(5)	78.8	31.0	59.6		WWTPs (Canada).	[43]
		(4)	*Data from bibliography*		> 90		WWTPs (China).	[34]
		(2)	0.1	0.4	−225	**−600**	WWTPs (Czech Republic).	[32]
		(2)	0.2	2.15	−975		WWTPs (Czech and Slovak Republics).	[35]
	**NTDA**	(4)	*Data from bibliography*		>90.0	>90.0	WWTPs (China).	[34]
	**DIE**	(1)	<0.8	<0.3	62.5	83.0	WWTPs (Switzerland).	[31]
		(3)	<0.8	<0.3	62.5		WWTPs (Switzerland).	[36]
		(2)	6.5	0.1	95.9		WWTPs (Czech Republic).	[32]
		(2)	6.4	0.30	95.3		WWTPs (Czech and Slovak Republics).	[35]
		(2)	3.9	4	−2.6	**−2.6**	WWTPs (Czech and Slovak Republics).	[35]
**Progesterone derivatives**	**NOMAC**	(2)	1.3	0.1	72.0	72.0	WWTPs (Czech Republic).	[32]
	**MEP**	(1)	6	3	50.0	52.6	WWTPs (Switzerland).	[31]
		(3)	6	3	50.0		WWTPs (Switzerland).	[36]
		(2)	0.1	0.04	46.8		WWTPs (Czech Republic).	[32]
		(2)	0.2	0.05	72.9		WWTPs (Czech and Slovak Republics).	[35]
		(2)	<0.02	0.23	**−1050**	**−725**	WWTPs (Czech Republic).	[32]
		(2)	0.19	0.95	**−400**		WWTPs (Czech and Slovak Republics).	[35]
	**MPA**	(1)	*Data from bibliography*		93.0	76.4	WWTPs (Switzerland).	[31]
		(2)	2.4	0.3	71.0		WWTPs (Czech Republic).	[32]
		(2)	2.2	0.22	90.1		WWTPs (Czech and Slovak Republics).	[35]
		(3)	3.1	0.2	85.6		WWTPs (Switzerland).	[36]
		(4)	*Data from bibliography*		24.0		WWTPs (China).	[34]
	**MGA**	(1)	*Data from bibliography*		99.6	78.2	WWTPs (Switzerland).	[31]
		(2)	6.4	0.3	95.3		WWTPs (Czech and Slovak Republics).	[35]
		(3)	<0.03	0.4	93.7		WWTPs (Czech Republic).	[32]
		(4)	*Data from bibliography*		24.0		WWTPs (China).	[34]
**Spironolactone derivative**	**DSP**	(1)	<4.0	<1.0	75.0	61.5	WWTPs (Switzerland).	[31]
		(2)	0.7	0.4	49.0		WWTPs (Czech Republic).	[32]
		(2)	3.5	0.1	88.2		WWTPs (Czech and Slovak Republics).	[35]
		(3)	<4.0	<1.0	75.0		WWTPs (Switzerland).	[36]
		(4)	*Data from bibliography*		42.0		WWTPs (China).	[34]

Note: Average values for WWTP_i_ and WWTP_e_ > 0 were calculated for Refs. [32,35,36,38,42]. Values of <0, corresponding to the last references are shown in bold. (1) Liquid chromatography with tandem mass spectrometry detection (LC-MS/MS); (2) liquid chromatography tandem atmospheric pressure chemical ionization/atmospheric pressure photoionization with hybrid quadrupole/orbital trap mass spectrometry operated in high-resolution product scan mode (LC-APCI/APPI-HRPS); (3) high-performance liquid chromatography coupled to triple quadrupole mass spectrometry (HPLC-MS/MS); (4) ultra-performance liquid chromatography coupled with tandem mass detection (UPLC-MS/MS); (5) triple quadrupole-linear ion trap mass spectrometer using the sMRM (scheduled multiple reaction monitoring) mode (TripleQuad-LIT-MS); (6) rapid resolution liquid chromatography/tandem mass spectrometry (RRLC-MS/MS).

**Table 4:** Main metabolisation organ and enzymes, elimination routes, and the number of active metabolites for the PGs referred to in this study. Data not available (n.a.).

**Testosterone derivatives** **(Gonanes)**	**PGs**	**Main Metabolization(s) Route(s)**	**Elimination Route(s)**	**Active Metabolites**	**References**
	GES	Liver. Metabolisation occurs by CYP3A4 via partial or total reduction of the A-ring.	Urine and faeces at a ratio of about 6:4.	n.a.	[58,59]
	LNG	Liver. Metabolisation by CYP3A4 and CYP3A5.	Urine (45%). Faeces (32%).	In sludge, LNG metabolites generate four active molecules.	[60,61]
	NET	Liver. NET is converted to LNG. Then, it follows the same metabolisation paths of LNG.	Urine (46%). Faeces (32%).	In sludge, NET metabolites generate four active molecules.	[61,62]
	ENG	Liver Metabolization by CYP3A4.	n.a.	n.a.	[63]
**Testosterone derivatives** **Estranes)**	**NTD**	Liver. Metabolisation occurs via partial or total reduction of the A-ring and oxidation by CYP3A4 and, to a much lesser extent, CYP2C19, CYP1A2, and CYP2A6.	Urine (50%). Faeces (20–40%).	The most known and active biologic metabolite is EE_2_.	[44,64,65,66]
	**NTDA**	Liver. NTDA is converted to NTD. Then, it follows the same metabolisation paths of NTD.	Urine (50%). Faeces (20–40%).	The most known and active biologic metabolite is EE_2_.	[44,64,65,66]
	**DIE**	Liver. Metabolisation by P450 enzymes.	Urine and faeces at a ratio of about 3:1.	The metabolites are all inactive.	[67]
**Progesterone derivatives**	**NOMAC**	n.a.	n.a.	n.a.	[68]
	**MEP**	Liver. Metabolisation occurs via partial or total reduction of the A-ring. May happen side-chain reduction, loss of the acetyl group, hydroxylation in the 2-, 6-, and 21-positions or a combination of these positions.	Urine.	More than ten active metabolites.	[69]
	**MPA**	Liver. MPA is converted to MEP. Then, it follows the same metabolisation paths of MEP.	Urine.	More than ten active metabolites.	[44,69,70]
	**MGA**	Liver.	Urine. Respiratory excretion. Fat storage.	n.a.	[71]
**Spironolactone derivative**	**DSP**	Liver. Metabolisation occurs by the opening of the lactone ring, known as M11, followed by the action of CYP3A4.	Urine (38–47%). Faeces (17–20%).	n.a.	[72]

**Table 5:** Data retrieved from “Web of Science Core Collection”, covering years from 2015 to 2021, concerning the effects of the synthetic PGs in fish. No data were available (n.a.) for NOMAC.

**Testosterone derivatives (Gonanes)**	**PGs**	**Structural or Functional Impact on Fish**	**References**
	**GES**	Induction of masculinisation in fathead minnow (*Pimephales promelas*).	[89]
		Reproductive disorders in zebrafish (*Danio rerio*).	[90]
		Induction of intersex in common carp (*Cyprinus carpio*).	[91]
		Masculinisation, potential reproduction reduction in mosquitofish (*Gambusia affinis*).	[92]
	**LNG**	Interfere with sex differentiation in zebrafish (*D. rerio*).	[93]
		Decrease of larval growth and expression of 20β-HSD and CYP19A1, FSH and 3β-HSD in fathead minnows (*P. promelas*).	[94]
		Inhibition of egg production in fathead minnows (*P. promelas*).	[95]
		Induction of precocious puberty in zebrafish (*D. rerio*).	[96]
		Alteration of fitness, ovary maturation kinetics and reproduction success in zebrafish (*D. rerio*).	[97]
		Change of the anal fin development and reproductive behaviour in mosquitofish (*Gambusia holbrooki*).	[98]
		Modification of oogenesis in fathead minnow (*P. promelas*).	[99]
		Induction of metabolic disorders in roach (*Rutilus rutilus*).	[100]
		Rise of nest acquisition success and loss of sperm motility in fathead minnow (*P. promelas*).	[101]
		Decrease of mature oocytes in zebrafish (*D. rerio*).	[97,102]
		Alteration of circadian gene regulation in zebrafish (*D. rerio*).	[103]
		Alteration of liver function in zebrafish (*D. rerio*).	[104]
		Transgenerational effects in inland silverside (*Menidia beryllina*).	[105]
		Decrease of post-hatch survival in zebrafish (*D. rerio*).	[106]
		Inhibition of swim bladder inflation in Japanese medaka (*Oryzias latipes*) embryos.	[107]
	**NET**	Transcriptional alterations in early development in zebrafish (*D. rerio*).	[108]
		Alteration of secondary sex characteristics, reproductive histology, and behaviours in mosquitofish (*G. affinis*).	[109]
		Transcriptomic and physiological changes in adult mosquitofish (*G. affinis*).	[110]
	**ENG**	Change of mating behaviour and reproduction in Endler’s guppies (*Poecilia wingei*).	[111]
**Testosterone derivatives (Estranes)**	**NTD**	Alteration of steroidogenesis in female fathead minnow (*P. promelas*).	[112]
		Alteration of sex differentiation in zebrafish (*D. rerio*).	[93]
		Alteration of circadian gene regulation in zebrafish (*D. rerio*).	[103]
		Alter the development of visual function in zebrafish (*D. rerio*).	[113]
		Induction of masculinisation and hepatopathological disorders in female mosquitofish (*G. affinis*).	[110]
		Alteration of mating behaviours, ovary histology and hormone production in zebrafish (*D. rerio*).	[114]
		Alters growth, reproductive histology, and gene expression in zebrafish (*D. rerio*).	[115]
		Thyroid endocrine disruption in zebrafish (*D. rerio*).	[116]
		Interfere with the HPG and the hypothalamic-pituitary-adrenal (HPA) axis in zebrafish (*D. rerio*).	[117]
		Neurodevelopmental effects in zebrafish (*D. rerio*).	[118]
		Hepatic injury in zebrafish (*D. rerio*).	[119]
	**NTDA**	Induction of developmental abnormalities in zebrafish (*D. rerio*).	[120]
	**DIE**	Minor transcriptional alterations in zebrafish (*D. rerio*) early life stages.	[121]
**Progesterone** **derivatives**	**NOMAC**	n.a.	n.a.
	**MEP**	Potential endocrine disruptor in fish.	[122]
	**MPA**	Reproductive disorders (gonadal histology) in zebrafish (*D. rerio*).	[123]
		Affects sex differentiation and spermatogenesis in zebrafish (*D. rerio*).	[124]
		Affects eye growth in zebrafish (*D. rerio*).	[125]
	**MGA**	Reproductive disorders of zebrafish (*D. rerio*).	[126]
		Alters ovary histology of zebrafish (*D. rerio*).	[127]
		Endocrine disruption in Chinese rare minnow (*Gobiocypris rarus*).	[128]
**Spironolactone derivative**	**DSP**	Alter plasma steroid levels and CYP_17_A_1_ expression in gonads of juvenile sea bass (*Dicentrarchus labrax*).	[129]
		Ethinylestradiol antagonist in zebrafish (*D. rerio*) embryos.	[130]
		Metabolic disorders in roach (*R. rutilus*).	[100]
		Together with GES induces intersex of common carp (*C. carpio*).	[91]

**Table 6:** Bioconcentration factor in fish plasma (BCF_FP_) and concentration in the plasma of a fish (C_FP_), which correspond to the human plasma therapeutical levels, and predicted effect concentration (PEC_w_) values. Data in bold are above PEC_w_, considering the average between the minimum and the maximal levels measured in surface waters (S_w_), WWTP influents (WWTP_i_) and effluents (WWTP_e_) presented in Table 2.

PGs	Log K_ow_	BCF_FP_	C_FP_ (ng/mL)	PEC_w_ (ng/L)	S_w_ (ng/L)	WWTP_i_ (ng/L)	WWTP_e_ (ng/L)
^a^ **GES**	3.26	32	1.0	31	10.8	10.9	10.8
^a^ **LNG**	3.48	46	2.4	52	**58.5**	**405**	19.5
^b^ **NET**	3.48	46	-	6.7	-	**11.2**	2.0
^a^ **ENG**	3.16	27	0.8	29	0.2	0.8	0.7
^a^ **NTD**	2.97	19	9.8	516	115	**568**	132
^a^ **NTDA**	3.99	108	9.8	91	0.3	10.5	0.62
^a^ **DIE**	2.34	7	85.2	12,171	1.2	6.4	2.2
^a^ **NOMAC**	3.55	52	7.2	138	0.1	1.8	0.1
^a^ **MEP**	3.50	47	1	21	0.7	3.0	1.5
^a^ **MPA**	4.09	128	1	8	0.2	4.1	0.3
^c^ **MGA**	3.20	29	-	-	10.0	6.5	30.0
^a^ **DSP**	4.02	113	30.8	273	2.2	3.5	0.5

^a^ Values of log K_ow_ and BCF_FP_ [6]; ^b^ Value of PEC_w_ determined for zebrafish in vivo, using an environmental relevant concentration of NET [139]; Value of log K_ow_ for ^c^ MGA [140].

**Table 7:** Assessment factors used for PNECs derivation [145].

Available Data	Assessment Factor (AF)
One short-term E(L)C_50_ from each of the three trophic levels (fish, crustaceans, or algae).	1000
One long-term NOEC assay (either fish, crustaceans, or algae).	100
Two long-term NOEC assays considering species from two trophic levels (fish and/or crustaceans and/or algae).	50
Three long-term NOEC assays considering species from three trophic levels (fish, crustaceans and algae).	10
Species Sensitivity Distribution (SSD) method	5–1
Field data or model ecosystems.	Evaluated on a case-by-case basis.

**Table 8:** Risk quotients (RQs) for 9 of the 12 PGs referred to in this study using the considering the average between the minimum and the maximal levels found in surface waters from 2015 to 2021. RQ values were not calculated for NTDA due to the absence of MEC and for NOMAC and MEP due to the lack of endpoint values for fish.

PGs	Endpoint Value (ng/L) Fish	PNEC (ng/L)	MEC (ng/L)	RQs	Risk	References
**GES**	EC50 = 10; AF = 1000	0.01	10.8	1078	High	[89]
**LNG**	NOEC = 0.42; AF = 50	0.01	59	6967	High	[43,95]
**NET**	LOEC = 6.0; AF = 1000	0.01	-	-		[90]
**ENG**	EC50 = 12,654; AF = 1000	12.7	0.2	0	Low	[146]
**NTD**	NOEC = 4; AF = 50	0.08	115	1438	High	[43,147]
**NTDA**	NOEC = 816; AF = 1000	0.8	0.3	0	Low	[43,147]
**DIE**	NOEC = 44; AF = 1000	0.04	1.2	36	High	[148]
**NOMAC**	NOEC = 1300; AF = 10	130	0.1	0	Low	[149]
**MEP**	-	-	0.7	-	-	-
**MPA**	NOEC = 342; AF = 50	6.8	0.2	0	Low	[43,123]
**MGA**	NOEC = 33; AF = 50	0.7	10.0	15	High	[43,126]
**DSP**	NOEC = 100; AF = 50	2.0	2.2	1.1	High	[43,150]
